# Diffuse Intrinsic Pontine Glioma: Molecular Landscape, Evolving Treatment Strategies and Emerging Clinical Trials

**DOI:** 10.3390/jpm12050840

**Published:** 2022-05-20

**Authors:** Sudarshawn Damodharan, Montserrat Lara-Velazquez, Brooke Carmen Williamsen, Jeffrey Helgager, Mahua Dey

**Affiliations:** 1 Department of Pediatrics, Division of Pediatric Hematology, Oncology and Bone Marrow Transplant, School of Medicine & Public Health, University of Wisconsin, Madison, WI 53792, USA; sdamodharan@uwhealth.org; 2 Department of Neurosurgery, School of Medicine & Public Health, University of Wisconsin, UW Carbone Cancer Center, Madison, WI 53792, USA; montselarav@gmail.com (M.L.-V.); williamsen@wisc.edu (B.C.W.); 3 Department of Pathology, School of Medicine & Public Health, University of Wisconsin, UW Carbone Cancer Center, Madison, WI 53792, USA; jhelgager@wisc.edu

**Keywords:** DIPG, high grade gliomas, childhood gliomas, *H3 K27M*

## Abstract

Diffuse intrinsic pontine glioma (DIPG) is a type of intrinsic brainstem glial tumor that occurs primarily in the pediatric population. DIPG is initially diagnosed based on clinical symptoms and the characteristic location on imaging. Histologically, these tumors are characterized by a heterogenous population of cells with multiple genetic mutations and high infiltrative capacity. The most common mutation seen in this group is a lysine to methionine point mutation seen at position 27 (K27M) within histone 3 (H3). Tumors with the *H3 K27M* mutation, are considered grade 4 and are now categorized within the *H3 K27*-altered diffuse midline glioma category by World Health Organization classification. Due to its critical location and aggressive nature, DIPG is resistant to the most eradicative treatment and is universally fatal; however, modern advances in the surgical techniques resulting in safe biopsy of the lesion have significantly improved our understanding of this disease at the molecular level. Genomic analysis has shown several mutations that play a role in the pathophysiology of the disease and can be targeted therapeutically. In this review, we will elaborate on DIPG from general aspects and the evolving molecular landscape. We will also review innovative therapeutic options that have been trialed along with new promising treatments on the horizon.

## 1. Introduction

Diffuse intrinsic pontine glioma (DIPG) is a primary central nervous system (CNS) malignancy that grows within the pons and predominantly affects the pediatric population. Filbin et al. found that these gliomas primarily contain oligodendrocyte precursor cells with a greater potential for proliferation and tumorigenesis than other similar cells or origin [1]. The tumor is characterized by its highly infiltrative nature and typical location within the brain. Although these tumors generally arise in the brainstem, invasion into the cerebellum and thalamus via white matter tracts and leptomeningeal dissemination is also seen. This led to the re-classification of DIPG encompassed within the diffuse midline glioma (DMG) category [2]. DIPG shares features with HGG (grade 4) or anaplastic astrocytoma (grade 3), and these gliomas clinically behave as high-grade malignant lesions with a dismal prognosis [3]. The current treatment approach to DIPG is radiation therapy; however, this is only palliative [4,5].

Historically, biopsy was typically not performed, due to the location; however, recent advances in surgical adjunct technology have made this safer, leading to a deeper understanding of this disease at a molecular level. DIPG was previously thought to resemble adult high-grade gliomas (HGG), but recent advancements have shown this not to be true. In 2021, the World Health Organization (WHO) updated the original brain tumor classifications, taking markers into consideration and classifying pediatric gliomas with a K27M mutation in histone H3 (3.1 or 3.3) as diffuse midline gliomas (DMG), *H3 K27*-altered [6,7]. Due to the poor prognosis of tumors with this mutation, they were assigned a grade 4 designation regardless of conventional histologic features [8]. This new classification now includes the majority of DIPGs as more than 80% of these tumors harbor this mutation [9].

In this review, we provide an overview of the epidemiology, clinical presentation, diagnosis and treatment of DIPG, with the primary focus on recent advances in the understanding of the molecular landscape related to disease initiation and prognosis, along with the pathways that are driving the next generation of therapeutic development. Finally, we outline promising current clinical trials that have been targeting molecular players important for the development and progression of DIPG.

## 2. Methods

We sought to write a comprehensive review article about DIPG with a focus on its molecular landscape along with current and upcoming treatment options. Articles utilized were predominantly from the last 25 years, with those dating beyond this excluded. However, older landmark DIPG studies along with published results from prior clinical trials were also included. Current ongoing clinical trials for the treatment of DIPG were assessed from www.clinicaltrials.gov and included those registered as of 28 January 2022.

## 3. Epidemiology

DIPG arises mainly in children, with a peak incidence at 6 to 9 years of age and with a similar distribution in males and females [4,10,11]. The exact incidence within the adult population is not known but is rare [12]. In the pediatric population, it accounts for about 15–20% of all brain tumors and represents 80% of those within the brainstem [13]. It is ranked as the second most common malignant brain tumor in children, with approximately 150–400 children per year being diagnosed within the United States [10]. The median survival for patients ranges from 8 to 12 months, with less than 10% of patient surviving past 2 years from their time of diagnosis [14].

## 4. Clinical Presentation

The clinical presentation of patients is wide varying and results from the compression or dysfunction of critical white matter tracks in the brainstem. Cerebellar dysfunction (dysarthria, ataxia, dysmetria), myelopathy (motor deficits, Babinsky sign, hyperreflexia) and cranial nerve deficits (diplopia and facial weakness) are the most common presenting symptoms [15,16]. Cranial nerve VI and VII are most commonly affected and specific to DIPG as they originate where the tumor typically infiltrates [17]. This leads to diplopia and a conjugate gaze palsy (i.e., abducens palsy) which is a common initial sign and is a poor prognostic factor [16]. Children present with these symptoms in less than a months’ time prior to their diagnosis [18]. Increased intracranial pressure and hydrocephalus occurs infrequently at disease presentation but can occur during the latter part of progression [2,19].

## 5. Imaging

**Figure 1:**
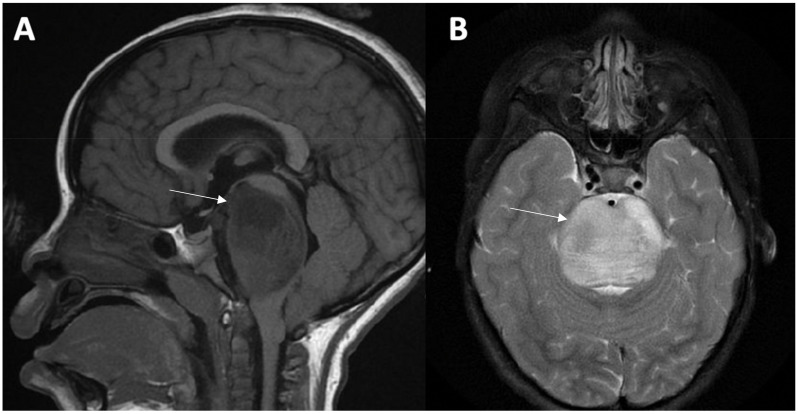
DIPG Imaging Findings. MRI of the brain with and without gadolinium in a child with an *H3 K27*-altered, DIPG. The imaging demonstrates a sagittal T1 hypointense (**A**) and axial T2 hyperintense (**B**) lesion with homogeneous enhancement along with obstruction within the fourth ventricle of the brain occupying almost half of the axial diameter.

The diagnosis is made based upon the patient’s clinical presentation along with radiographic findings of the tumor within the pons. Magnetic resonance imaging (MRI) with and without contrast is the imaging modality of choice for the diagnosis [20,21]. The classic MRI findings include T1-hypointensity and T2-hyperintensity with ill-defined margins along with minimal contrast enhancement around areas of necrosis or inflammation [21]. The tumor is generally centered within the pons and at the time of diagnosis can occupy greater than 50% of the axial diameter [20,21] (Figure 1).

Advanced MRI sequences have also been studied to see if they may play a role in aiding the diagnosis or management. One of these techniques is diffusion tensor imaging (DTI), which has been considered to aid in defining cortical white matter tracts from brain neoplasms and may even help differentiate DIPG from other neoplasms [22,23,24]. Along with this, a recent report suggested that the apparent diffuse coefficient (ADC) may play a prognostic role as patients with a higher value showed improved survival [23].

Positron emission tomography (PET) is also considered to aid in the evaluation. In the preclinical setting, a group at Memorial Sloan Kettering characterized a PET probe targeting PARP1, which is highly expressed in DIPG compared to normal tissue in a murine model [25]. They demonstrated that this mode of imaging can quantify the tumor burden in a murine model and could aid in the diagnosis and future monitoring of treatment response in clinical trials [25]. New approaches using PET tracers can potentially identify relevant biomarkers without the need for invasive procedures, allowing for more personalized treatment and improved quality of life [25].

## 6. Histologic Characteristics

Historically, biopsy was not undertaken, due to the tumor location and increased morbidity associated with surgical intervention. However, increasing advances to better understand the molecular environment along with improvements in surgical techniques have helped move this towards the standard approach [26,27,28].

**Figure 2:**
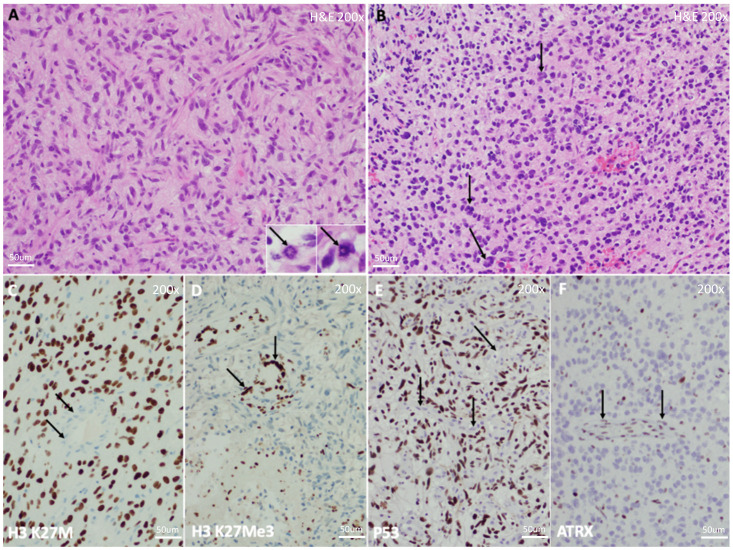
Histology of *H3 K27*-Altered DIPG. Histology of Diffuse Midline Glioma, *H3 K27*-Altered, WHO Grade 4, the most common tumor comprising DIPG. (**A**) Microscopy demonstrates a high grade, infiltrative glioma with large, atypical, and hyperchromatic nuclei in a fibrillary background. Though high-grade features, including mitotic figures (inset, arrows), are not requisite for the diagnosis, they are generally easy to detect. Microvascular proliferation and necrosis may be variably present and may not be represented on small biopsies. (**B**) Another tumor from a different patient, demonstrating somewhat variable histology, in this case with particularly hyperchromatic, round, and atypical nuclei with frequent multinucleation (arrows). (**C**) Immunohistochemistry demonstrates diffuse nuclear positivity with an antibody detecting the *H3 K27M* mutation, which is diagnostic for this tumor. Note adjacent negativity within blood vessels and infiltrated non-neoplastic brain parenchyma (arrows), serving as a negative internal control. (**D**) H3K27Me3 immunostaining, which detects trimethylation at the K27 residue, is always lost secondary to the K27M mutation and should be used as a confirmatory stain in making the diagnosis, though DNA sequencing is the diagnostic gold standard. Adjacent non-neoplastic tissue retains trimethylation, and stains positive (arrows). *TP53* and/or *ATRX* mutations are frequent within these neoplasms, which generally manifest as diffuse nuclear positivity for the *TP53* protein by immunostaining (**E**), or loss of nuclear positivity for the *ATRX* protein (**F**). Note negative and positive non-neoplastic tissue, respectively, serving as internal controls (arrows).

Histologically, DIPG has conventionally been characterized as a heterogeneous disease with a varied spectrum of morphologies and grades [2,29] (Figure 2). Yoshimura et al. reviewed the autopsies of 33 pediatric patients with brainstem gliomas and reported that 29 of them had glioblastoma (GBM, WHO grade 4) histology and 2 anaplastic astrocytoma (AA, WHO grade 3) histology [2]. An even larger autopsy-based assessment demonstrated 42 cases of GBM, 18 AA, 8 low-grade glioma (LGG, WHO grade 1 and 2) and 2 with histology consistent with primitive neuroectodermal tumor (PNET, WHO grade 4) [30]. The 2018 collaborative report from the International and European society for Pediatric Oncology (SIOP) DIPG Registries showed a very similar histologic variation in their review of 288 biopsy and 76 autopsy samples [29].

However, these studies found no difference in overall survival based on the histological grade of the tumor. Both the studies conducted by Buczkowicz et al. and the SIOP DIPG Registries showed that patients with a low-grade lesion do just as poorly as those with a high-grade [29,31,32]. More recent studies analyzing the molecular profiles of DIPG found that most harbor a mutation in histone H3 and that they are more aggressive and have worse outcomes compared to their non-mutant counterparts, regardless of histology [33]. This finding has helped lead to the new WHO classification of *H3 K27*-altered brainstem and midline tumors as a separate, grade 4 pathologic entity that has become the clinical standard for the diagnosis.

## 7. Molecular Pathways

**Figure 3:**
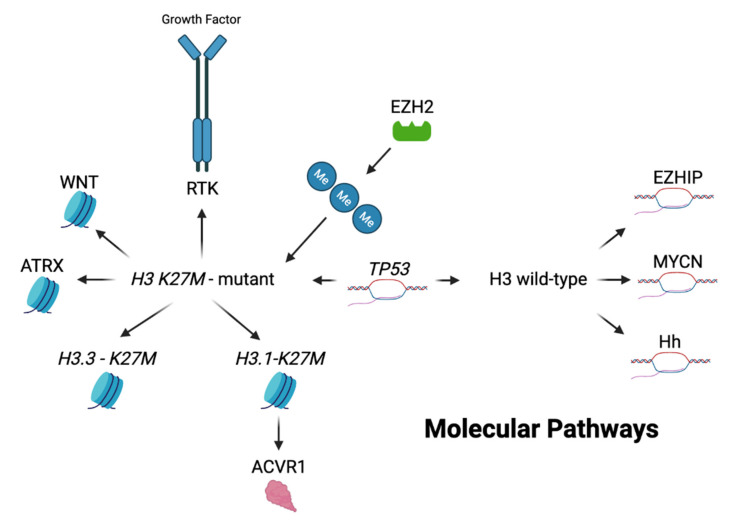
Genetic landscape of DIPG. Here outlines a brief overview of the genetic landscape and molecular pathways of *H3 K27M*-mutant and non-mutant pediatric gliomas. The *H3 K27M* mutation can be divided into two particular genetic alterations—*H3.3* on the *H3F3A* gene and the *H3.1* on the *HIST1H3B* gene. Of these, the *H3.3* mutation is more commonly altered in *H3 K27M*-mutant pediatric gliomas. Enhancer of zeste homolog 2 (*EZH2*) is closely associated with *H3 K27M*-mutant gliomas leading to hypermethylation, increased mutant gene expression and tumor formation. The receptor tyrosine kinase (RTK) pathway is also closely associated with *H3 K27M*-mutant gliomas. They play an integral role in tumor development and cell proliferation via their affects primarily on platelet derived, epidermal and fibroblast growth factor receptors. Mutations within the WNT pathway lead to increased levels of β-catenin, causing an over proliferation of malignant cells and has shown to occur concurrently with the *H3 K27M* mutation in a preclinical murine model. Histone chaperone alpha-thalassemia/mental retardation syndrome X-linked (*ATRX*) has a high co-occurrence with *H3 K27M*-mutant gliomas and contributes to tumor development by destabilizing telomeres and altering gene expression at a molecular level. *ACVR1* mutated gliomas are more prominently found with the *H3.1 K27M* mutation, leading to increased tumor proliferation via dysregulation of the BMP/SMAD pathway. This mutation tends to lead to tumor formation at an earlier age along with increased overall survival. *TP53* alterations have been shown to occur in high frequency with the *H3 K27M*-mutation but are also present on H3 wild-type tumors. Abnormal *TP53* function leads to increased DNA and protein instability causing decreased apoptosis and increased tumor development. Alterations to the *MYCN* and Hedgehog (*Hh*) signaling pathways occur more frequently in H3 wild-type tumors. *MYCN* alterations lead to overexpression of this transcriptional factor resulting in tumor formation via DNA hypermethylation and chromosomal rearrangement. Disturbances to the Hh pathway led to ventral pontine hyperplasia in a murine preclinical DIPG model and are proposed to play a role in tumor formation independently of the *H3 K27M* mutation. Enhancer of zeste homologs inhibitory protein (EZHIP) overexpression is present in the majority of H3 wild-type tumors and is characterized by the overexpression of the CXorf67 gene. This overexpression mimics the amino acid sequence present within *H3 K27M*-mutant gliomas, leading to global hypomethylation. Direct inhibitors of EZHIP have not yet been found but are currently being studied as a primary target for H3 wild-type gliomas. Image created with BioRender.

Due to the high rates of therapeutic failure, DIPG was initially thought to have similar genetic profiles as adult high-grade gliomas, and thus were treated in a similar manner [34]. However, recent genome-wide analysis has shown unique molecular entities which have been linked with these gliomas (Figure 3). The tumor is primarily categorized as being *H3 K27M*-mutant or non-mutant, with the majority harboring the mutation. The new 2021 WHO classification of CNS tumors has listed these under the *H3 K27*-altered category with four distinct primary subtypes including *H3.3 K27M*-mutant, *H3.1 K27M*-mutant, H3-wildtype and EGFR-mutant [35]. Other pathways are also hypothesized to play a role in tumor development [15]. Here, we outline molecular alterations found within DIPG and their occurrences within the *H3 K27M*-mutant or non-mutant groups.

### 7.1. H3 K27M

**Figure 4:**
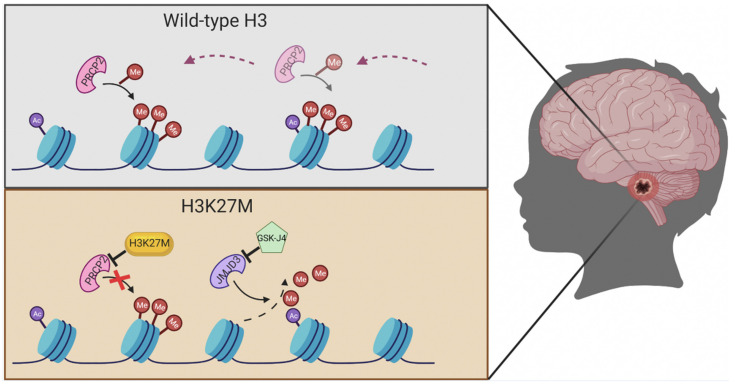
Significance of *H3 K27M*. Overall, 80% of all DIPG exhibit a missense mutation at histone *H3 K27M*. This mutation is a substitution of lysine with methionine in the position 27 (K27M). Histone H3 is a checkpoint control of transcriptional regulation in the synthesis phase of DNA. When mutated, H3 K27 trimethylation is ablated resulting in a repression of the polycomb repressive complex 2 target genes, which can lead to chromatin disaggregation and cellular aneuploidy. The prognosis for these *H3 K27M*-mutant pediatric gliomas is very poor and universally fatal. Image created with BioRender.

The *H3 K27M*-mutant subgroup exhibits a missense mutation in histone 3 with the substitution of lysine (K) for methionine (M) at position 27 (Figure 4). Two variations of this mutation include *H3F3A* (H3.3) or *HIST1H3B/C* (H3.1) [36,37]. This genetic alteration is present in approximately 80% of all DIPG [28]. The *H3 K27M* mutation has also been associated with midline gliomas. In 2016, the WHO classified these tumors into the pathologic entity “diffuse midline glioma, *H3 K27M*-mutant”, a grade 4 malignancy regardless of conventional histologic criteria [36]. The 2021 WHO classification of tumors of the CNS reclassified *H3 K27M*-mutant DIPGs under the “diffuse midline glioma, *H3 K27*-altered” category [35]. Although the exact role of the *H3 K27M* mutation in tumor development is not fully understood, the mutation is linked to a worse prognosis [36,38]. DIPG itself is universally fatal given the location, but studies show that those that harbor the *H3 K27M* mutation have an even shorter overall survival (OS) with those harboring H3.3 faring worse than H3.1 [36]. A meta-analysis performed by Mackay and colleagues in 2017 showed that the survival time for *H3 K27M*-mutant DIPG was 2.5 months less that the wild type [36]. Among the *H3 K27M*-mutated midline gliomas, survival is also affected by the neuroanatomical location. In 2018, Karreman et al. showed that pediatric patients with thalamic *H3 K27M*-mutated midline gliomas survived longer than those with DIPG or tumors within the spinal cord [39].

### 7.2. Histone Chaperone Alpha-Thalassemia/Mental Retardation Syndrome X-Linked (ATRX)

The *ATRX* complex mutation has a high co-occurrence with the *H3 K27M*-mutant tumors [35,40]. The depletion of the complex is hypothesized to contribute to tumor development by causing destabilization of telomeres and altering gene expression in conjunction with the *H3 K27M* mutation [33,35,40]. There are also strong co-occurrences of the *TP53*, platelet-derived growth factor receptor (PDGFRA) amplification and *ACVR1* genetic alterations within the *H3 K27M*-mutated category [35]. The clinical significance of these co-occurrences remains unknown, with further research ongoing to better understand the varying genetic landscape of *H3 K27M*-mutant tumors.

### 7.3. Enhancer of Zeste Homolog 2 (EZH2)

Recent data suggests that *EZH2* activity plays a role in the growth of *H3 K27M*-mutant DIPG cells in in vivo mouse models [41]. *EZH2* is involved in many cellular processes including cell cycle progression, proliferation and apoptosis [41,42]. The mutant *H3 K27M* binds to *EZH2*, which interferes with the methyltransferase activity resulting in hypermethylation and increased tumor formation [41,42]. *EZH2* inhibitors have shown promising results in preclinical models in combination with other targets for treatment of DIPG, but more research is needed to better translate this data into human applicability [42].

### 7.4. Receptor Tyrosine Kinase (RTK)

RTKs are transmembrane protein receptors that contain intrinsic enzymatic activity, and they play a critical role in signaling pathways that include cell proliferation, differentiation and survival. RTKs include platelet-derived growth factor receptors (PDGFR), epidermal growth factor receptors (EGFR) and fibroblast growth factor receptors (FGFR). Various amplifications and mutations to components within the RTK-RAS-PI3K pathway are seen in up to 60% of DIPG and occur frequently with the *H3 K27M*-mutant group [43]. EGFR-mutant gliomas have more recently been classified per the WHO as a distinct subtype of *H3 K27*-altered gliomas with primary abnormalities occurring within the EGFR oncogene on chromosome band 7 [35]. It is thought that potential treatments targeting this specific EGFR alteration may benefit these patients, though further research is needed. Histone H3.3 glycine 34 to arginine/valine (G34R/V) mutations encompass another subset of pediatric HGG with about half of these bearing activating PDGFR mutations [44]. Unlike the *H3 K27M*-mutant gliomas, these primarily occur within the cortex and other superficial areas within the brain with a GABAergic inhibitory interneuron cell of origin [44].

### 7.5. WNT

The *WNT* pathway is known to promote cell growth, survival and decreased apoptosis [45,46]. Mutations in this pathway results in increased levels of β-catenin, which is a key protein in *WNT* signaling and leads to an over proliferation of cells [45]. In an orthotopically xenografted *H3 K27M*-mutant murine model, the DIPG cells demonstrated active WNT signaling suggesting their co-occurrence [45]. The oncogenesis and severity of those with DIPG and mutations altering the WNT pathway have not been well described but is hypothesized to have a less severe phenotype as seen in the WNT subgroup of pediatric medulloblastoma and other HGGs [47,48].

### 7.6. Activin A Receptor, Type 1 (ACVR1)

*ACVR1* is a bone morphogenic protein (BMP) receptor which belongs to the TGF-beta signaling family [32,49,50,51]. It binds to many ligands and is crucial for signaling resulting in phosphorylation and activation of growth-promoting genes through SMAD transcription factors [52]. Certain mutations within *ACVR1* are present in the molecular make-up of DIPG. Somatic mutations R206H, R258G, G328E/V/W and G356D within *ACVR1* have been found in up to 25% of DIPG in retrospective analysis [50]. In normal conditions, *ACVR1* helps with myelination within the CNS [53]. When mutated, it encodes a serine/threonine kinase (ALK2) receptor with enhanced sensitivity to the ligand activin A, resulting in dysregulation of the BMP/SMAD pathway and increased tumor proliferation [49,53]. There is also a co-occurrence with the *H3 K27M* mutation with both being present in up to 22% of DIPG and the majority of these associated with the *H3.1* variety [50,51]. The mutation also tends to an earlier age of tumor development with a median of 5 years at diagnosis along with a slightly increased OS of 15 months [53].

### 7.7. Tumor Protein p53 (TP53)

*TP53* is a tumor suppressor gene that controls cellular functions including cell cycle senescence, apoptosis and metabolism. Abnormal *TP53* function in cancer cells can lead to increased DNA and protein instability causing decreased apoptosis [54,55]. This mutation is present in up to 75% of DIPG samples along with an increased co-occurrence with the *H3 K27M* mutation [54,55,56]. In a retrospective analysis, *H3 K27M*-mutant and *TP53*-mutant DIPG had increased RT resistance, enhanced tumor aggressiveness and worse OS in comparison to patients without the mutations or with only one mutation present [55]. Though the *H3 K27M* mutation is likely the primary oncogenic driver, the presence of both mutations led to worse clinical outcomes and proposes possible synergism with a more aggressive phenotype. This shows the multifactorial molecular mechanisms that make up DIPG and contribute to its fatal nature.

### 7.8. MYCN

The transcriptional factor *MYCN* and its protein target *PVT1* are also overexpressed and amplified, resulting in tumor initiation, progression and recurrence [57]. The DIPG *MYCN* molecular subtype is characterized by DNA hypermethylation and chromosomal rearrangement resulting in aneuploidy [58,59]. It is proposed that the *MYCN* pathway may be induced by the *H3 K27M* mutation, but the pathways are otherwise independent [33,60,61]. The overall prognosis of *MYCN* mutated DIPG in comparison to others is not completely well understood at this time. Given *MYCN*’s association with other adult and pediatric high-grade gliomas, it is proposed that the mutation carries a more severe phenotype [62].

### 7.9. Hedgehog (Hh) Signaling

Hh pathways play a major role in the regulation of processes such as cell proliferation and stem cell maintenance [6,63]. Abnormal signaling in the Hh pathway has shown ventral pontine hyperplasia within pre-clinical murine models [7,63]. Monje et al. showed that the upregulation of the Hh pathway may in fact play a role in DIPG tumor formation in a portion of DIPG patients independently of the presence of *H3 K27M*, though it is possible that a second stimulus may still be needed for tumor development [63].

### 7.10. Enhancer of Zeste Homologs Inhibitory Protein (EZHIP)

H3 wild-type glioma are characterized predominantly by the overexpression of the *CXorf67* gene which encodes *EZHIP* [64]. Given its high rate of occurrence, *EZHIP*-overexpressing DMG has been given its own classification in the latest WHO classification system. The c-terminal peptide in *EZHIP* mirrors that seen in the *H3 K27M* mutation leading to hypomethylation via reduced histone methyltransferase activity [64]. This alteration tends to lead to a slightly prolonged OS, though remains fatal [35]. Unfortunately, there is not yet a direct inhibitor that has been identified for this alteration, but further studies are ongoing to further address this.

## 8. Clinical Management

### 8.1. Surgical Intervention

Surgical management has been a topic of debate for many years with the initial thought to forego biopsy or further interventions given the tenuous location and limited therapeutic options [26,28,41]. However, as molecular analysis has continued to progress, so has the mindset of obtaining a biopsy at the time of diagnosis. Conventional needle biopsies had been avoided in the past, due to the difficulty in determining a safe trajectory [65]. Stereotactic biopsy is now being utilized more commonly along with newer navigation technologies and high-resolution imaging modalities [28]. A meta-analysis conducted by Hamisch et al. in 2017 retrospectively reviewed 735 brainstem tumor patients who underwent stereotactic biopsy and found a diagnostic yield of 96.1% with only a 0.6% mortality rate [26]. This shows that stereotactic biopsy is a safer procedure than previously thought. Additionally, others have similarly shown that brainstem biopsies can be obtained safely [66,67,68]. A meta-analysis of 13 studies that performed stereotactic biopsies of brainstem lesions in 381 patients showed a 96% diagnostic yield with a low rate of morbidity and mortality [68]. This highlights that pontine biopsy can be carried out safely at experienced medical centers.

### 8.2. Liquid Biopsy

Liquid biopsy is a newer technique that has been trialed for various malignancies. This is a non-invasive technique that involves obtaining biofluids (i.e., blood, CSF, urine or saliva) to detect circulating tumor DNA (ctDNA) or the tumor cells themselves [69]. Huang et al. used this approach in a cohort of patients that included pediatric DIPG to detect H3 mutations within the CSF via tumor derived cells [70]. Though this appeared to be a viable option to detect the molecular rearrangement, the sensitivity was poor [70]. Pages et al. prospectively collected blood, urine and CSF samples from 258 pediatric patients with various brain tumors and found that CSF contained the most ctDNA, though again showed the overall sensitivity to be poor [71]. Currently, obtaining a lumbar puncture at the time of diagnosis is not the standard of care, but the ability to test for ctDNA that has the ability to detect the *H3 K27M* mutation does appear to be promising with further validation needed [40].

### 8.3. Radiation Therapy

Radiation therapy (RT) is the standard treatment of DIPG; however, it is only palliative [65]. RT is expected to increase survival for patients by about 3 months on average [72,73]. The current standard radiation scheme involves 54–60 Gy to be given in fractionated doses (typically 1.8–2 Gy daily) over a 6-week span [74]. This scheme was first developed in 1988 by Freeman et al., and many further studies have looked to see if this could be improved upon [4]. A recent systematic review of the role of RT in the management of DIPG was done by Gallitto et al. This showed that the median OS of patients who received either the standard or modified hyper- or hypo-fractionated RT remained unchanged between 1988 and 2017 [75].

All the reviewed studies used photon beam RT. Proton beam RT is a newer modality that has the ability to more precisely target the tumor and thus reduces radiation exposure and potential damage to healthy tissue. It has been hypothesized to possibly reduce radiation toxicities by delivering therapy more precisely to the affected areas, though it has shown no increased survival for DIPG [75,76]. Additionally, proton beam RT is not widely available and has shown similar rates of radiation necrosis to that of conventional photon therapy [76].

Janssens et al. evaluated a hypo-fractionated RT compared to the conventional and found no impact on OS [77]. However, giving the RT at hypo-fractionated dosing (39 Gy in 13–16 fractions) did decrease the total number of sessions, which had an impact on the amount of time the patient spent in the hospital along with the associated social and emotional burdens [77]. A hyper-fractionated approach (78 Gy) was not advantageous without any survival benefit [14].

Following RT, tumor recurrence is universal and occurs approximately 6 months after treatment [78]. For patient with recurrent or progressive tumors, salvage re-irradiation is an option to help with controlling tumor progression and neurologic sequalae [78]. Gallitto et al. showed that reirradiation prolonged survival by approximately 10 months, though this came with significant neurologic deficits [75]. A multi-collaborative analysis lead by SIOP showed similar results that reirradiation slowed tumor progression but had significant risks and burdens [79]. Based on these studies, reirradiation is not typically recommended (Table 1).

### 8.4. Chemotherapy

Many chemotherapeutic agents have been tested as either a single modality treatment or in combination with radiation. Unfortunately, very few have led to any significant improvement in survival beyond what has been shown with radiotherapy alone [13].

As with many other malignant gliomas, DIPG does not show a significant response to the oral chemotherapy agent temozolomide (TMZ) which can cross the blood–brain barrier (BBB). A recent study by Izzuddeen et al. in 2019 tested the use of TMZ concurrently and following conventional RT versus RT alone in 33 patients [80]. The median OS for the experimental group was 12 months, with only a month extension over the conventional treatment group and significant hematologic toxicities associated with TMZ [80]. In 2011, Cohen et al. explored the use of TMZ as concomitant therapy to radiation in patients with newly diagnosed DIPG and found very similar results with no significant improvement in event-free survival (EFS) or OS [81]. The results of a United Kingdom phase II trial (CNS 200704) involving 43 patients also showed no benefits with prolonged regimens of TMZ given along with RT to patients who received RT alone [82]. It is hypothesized that the lack of BBB disruption in DIPG compared to other gliomas may account for the limited penetration of this agent and thus the lack of response to treatment [83].

Other chemotherapeutic approaches with proven successes in other neuro-oncological malignancies have also been tested with limited success. These agents include, but are not limited to, etoposide, carboplatin, vincristine, methotrexate, busulfan and bevacizumab. The majority of these agents are associated with poor clinical benefits along with significant adverse toxicities [84]. A trial by Frappaz et al. looked at the utility of administering pre-radiation cisplatin and methotrexate [72]. This showed an increased median survival up to 17 months with the addition but came with higher toxicity and infection rates that resulted in prolonged hospitalizations in the chemotherapy-treated group compared to RT alone [72]. Hargrave et al. conducted a meta-analysis of 29 clinical trials involving 973 patients and concluded that there is no improvement of outcomes with chemo-radiotherapy before, during or after conventional RT [74].

The synergistic effects of multiple chemotherapeutics have also been tested to target multiple pathways. Crotty et al. retrospectively examined the survival of patients treated with a combination regimen of TMZ, irinotecan and bevacizumab at Seattle Children’s Hospital from 2009 to 2018 [85]. This showed an increased 1-year OS in those treated with bevacizumab (80%) over the historical group (45.3%) but also suffered significant toxicities following systemic chemotherapy [85]. The Children’s Oncology Group (COG) ACNS0423 clinical trial looked at TMZ and radiation followed by lomustine and showed marginal benefits in the 3-year EFS [86]. The COG ACNS0822 trial compared the treatment outcomes of vorinostat, bevacizumab or TMZ given concomitantly to conventional RT followed by bevacizumab and TMZ for maintenance therapy [87]. This showed a 1-year EFS of 36.5%, 10.1% and 10.2% for vorinostat, bevacizumab or TMZ [87]. Significant hematologic and systemic toxicities were seen along with increased incidences of intracranial hemorrhage [87]. The HERBY clinical trial (NCT01390948) evaluated the addition of bevacizumab to conventional RT plus TMZ [88]. This showed that the addition of bevacizumab to RT and TMZ did not improve EFS [88].

Overall, chemotherapeutic agents do not appear to reach the tumor in high enough concentrations to be effective because the BBB appears well preserved [11,89]. A trial of high-dose myeloablative chemotherapy with autologous stem cell rescue was attempted to overcome this, but it was unsuccessful [84]. The outcomes of this study only showed a median survival time of 10 months for its patients without any significant benefit compared to RT alone [84]. This suggests that DIPG lacks response to both traditional and highly cytotoxic chemotherapeutic options (Table 2).

## 9. Ongoing Investigations and Future Considerations

Many ongoing investigations are looking into other avenues of treatment for DIPG. Currently, there are 99 clinical trials that are identified and available for view within the portal of ClinicalTrials.gov. Of these, only 10 trials have results readily available for review (Table 3). In review of these trials, none showed significant clinical benefits in OS, progression-free survival (PFS) and objective response. The modalities of treatment included immunotherapeutic options and chemotherapeutic regimens with or without RT [90]. Despite this, it is promising to see the various treatment modalities being trialed and researched.

### 9.1. Immunotherapy

Immunotherapeutic options have shown promising results in the treatment of various CNS malignancies, including other HGGs. These agents include chimeric antigen receptor (CAR) T cells, checkpoint blockade, vaccine and oncolytic viral therapies. The thought is to utilize immunotherapeutic options to either boost or alter a patient’s immune system to combat the tumor (Table 4).

The tumor microenvironment has been evaluated in many recent studies and is neutral in regard to an inflammatory or immunosuppressive state [97]. With common neoantigens that have been found, the goal is to target them with treatments that will drive T cells to halt tumor progression. CAR T cell therapy has been promising in this respect because of the neutral microenvironment, which would hinder the inflammatory or anticancer effects [98,99,100]. In preclinical studies, CAR T cells targeting GD2, a disialoganglioside highly expressed in *H3 K27M*-mutated gliomas, have shown promising results by eliminating the tumor in a xenograft model [98,99]. Currently, clinical trials utilizing GD2 specific CAR T-cell therapy are ongoing to determine its efficacy and safety. Majzner et al. recently published preliminary results of their phase 1 clinical trial utilizing a GD2 CAR approach for *H3 K27M*-mutant DIPG/DMG with three out of four patients showing initial radiographic and clinical benefits, though all did succumb to their disease [101]. These results highlight the possibility of CAR T therapy as a viable approach to target this malignancy. Additionally, B7H3 has been an additional target for therapy with ongoing trials also investigating its efficacy [100]. A limiting factor is the inherent inflammation associated with the anti-tumor effect of this therapy and the location within the CNS that would be affected. Further research continues to be done to find additional targets and neoantigens to be used. Although promising, the safety of CAR T therapy must be considered along with insights on how to prevent any associated toxicity.

Viral therapy has also emerged as a promising option. An oncolytic adenovirus has shown promise in the treatment of HGGs including DIPG in children. A phase I trial by Lang et al. in 2018 aimed to provoke an immune response by injecting DNX-2401 oncolytic adenovirus directly into 25 patient tumors [102]. The study observed that 18 of these patients, including those with DIPG, showed tumor shrinkage and 5 of these patients survived for more than three years after treatment [102].

ONC201 is an immunostimulatory small molecule antagonist dopamine receptors (DRD2/3) that has been reported in a range of malignancies including *H3 K27M*-mutant DIPG/DMG [103]. In preclinical murine models, this has been shown to double the OS [104]. Currently, there is an ongoing phase 2 clinical trial that looks to assess its efficacy further in pediatric *H3 K27M*-mutant gliomas.

Although it is too early to say if immunotherapeutic options will be successful, it does represent an innovative approach with potential to improve current outcomes. Immunotherapy-induced inflammation remains a concern with ongoing concurrent treatments to combat this. [99].

### 9.2. Epigenetic Modifying Agents

Epigenetic changes play a role in tumor development. Modifiers such as histone deacetylase (HDAC) inhibitor, panobinostat, has been utilized as a multi-HDAC inhibitor to increase H3 acetylation and *H3 K27M* methylation to reduce oncogenesis and tumor formation [105,106]. Panobinostat has shown promising results in pre-clinical *H3 K27M*-mutant glioma models. It does have a narrow therapeutic index that can cause dose limiting toxicities which has limited its efficacy [60]. Currently, panobinostat is being tested in clinical trials to determine its efficacy as a monotherapy or in combination with other treatments [90]. EZH2 is also highly expressed on *H3 K27M*-mutant gliomas [42,107]. Mohammed et al. showed that tazemetostat, an EZH2 inhibitor, does have anti-tumor properties in a pre-clinical DIPG model and could be efficacious in treatment but further studies are needed [107]. Bromodomain and extraterminal (BET) family proteins regulate expression of various oncogenes and are involved in cell cycle arrest that can lead to tumor proliferation [108]. JQ1 is a BET inhibitor that has shown inhibition of DIPG growth in preclinical models when combined with other agents such as EZH2 and other small molecule antagonists, though has yet to be translated into clinical trials [42,109]. Studies are also ongoing to find ways to limit the impact of *ACVR1* mutations that are observed. An ALK2/BMP type 1 receptor kinase inhibitor has been studied in pre-clinical models for other disease processes (i.e., fibrodysplasia ossificans progressiva) associated with *ACVR1* with good results. The effects of this for DIPG development remain unknown, with further research needed [27]. Finally, a JMJD3 inhibitor (GSKJ4) has also shown anti-tumor properties in pre-clinical *H3 K27M*-mutant studies [21,110]. JMJD3 is as a key enzyme in *H3 K27M* demethylation which leads to suppression of the PCR2 target genes, causing chromatin disaggregation, cellular aneuploidy and tumorigenesis [110,111]. Utilizing GSKJ4 treatment, up to 50% growth inhibition of *H3 K27M*-mutant cells was seen in pre-clinical studies with further translational work needing to be done to asses this as a viable treatment [21].

### 9.3. Convection Enhanced Delivery (CED)

CED is a methodology to overcome the difficulty of delivering drugs through the BBB. It is a relatively low-risk method of distributing therapeutic drugs directly into and around the brain tumor stereotactically via hydraulic pressure through catheters [112]. It is designed to direct drugs to a specific region of the brain in effective concentrations. Various agents are being tested for stability and effectiveness when used with CED. A phase I trial used convection enhanced delivery to deliver a radioimmunotherapy agent, omburtamab, that targets the B7-H3 antigen to 28 children [113]. The first results found an OS of 15.3 months with three of the children surviving for over three years [113]. In another recent trial by Szychot et al., nine children were treated with CED infusions of carboplatin and sodium valproate [114]. This study also showed a prolonged survival for patients [114]. Because CED concentrates drugs in a specific region in the brain, systemic toxicity is limited [115]. There are limitations with backflow, so new cannula designs are being studied to allow for higher flow rates without increased reflux [116]. Newer capabilities of PET are also being studied to better monitor the distribution of the drugs administered [117]. CED is only performed in a single session currently, but additional research is needed to determine the efficacy of delivering multiple/continuous doses of chemotherapy [112].

## 10. Conclusions

In summary, DIPG continues to have a poor prognosis, but advances are ongoing. While the diagnosis is typically solely made based upon radiographic evidence on MRI, the need for biopsy sampling has become more evident to advance our understanding of the disease. Radiation therapy continues to remain the standard treatment option. The current environment points towards epigenetic modifiers and immunotherapy being the next wave of therapies along with advancing surgical and therapeutic barriers to bypass the BBB. Future therapeutic approaches should account for the molecular subgroups, specifically the *H3 K27*-altered that has been given its own designation and comprises the majority of DIPG.

## Figures and Tables

**Table 1:** Active clinical trials for children with diffuse intrinsic pontine glioma involving radiation therapy as found on www.Clinicaltrials.gov as of 12 January 2022.

NCT Number	Title	Intervention	Location
NCT03126266	Re-irradiation of Progressive or Recurrent DIPG	Radiation: re-irradiation	Alberta Children’s Hospital, Calgary, Alberta, Canada
NCT04250064	A Study of Low Dose Bevacizumab with Conventional Radiotherapy Aline in DIPG	Drug: Bevacizumab Injection Radiation: Ultra-low-dose RT	Tala Memorial Hospital, Mumbai, Maharashtra, India
NCT03605550	A Phase 1b Study of PTC596 in Children with Newly Diagnosed DIPG and High-Grade Glioma	Drug: PTC596 Radiation: Radiotherapy	Children’s Hospital Colorado, Aurora, Colorado, United States Children’s National Medical Center, Washington, District of Columbia, United States Ann & Robert H. Lurie Children’s Hospital of Chicago, Chicago, Illinois, United States Dana-Farber Cancer Institute, Boston, Massachusetts, United States Duke University Medical Center, Durham, North Carolina, United States Cincinnati Children’s Hospital Medical Center, Cincinnati, Ohio, United States Nationwide Children’s Hospital, Columbus, Ohio, United States The Children’s Hospital of Philadelphia, Philadelphia, Pennsylvania, United States Texas Children’s Hospital, Houston, Texas, United States Seattle Children’s Hospital, Seattle, Washington, United States
NCT04049669	Pediatric Trial of Indoximod with Chemotherapy and Radiation for Relapsed Brain Tumors or Newly Diagnosed DIPG	Drug: Indoximod Radiation: Partial Radiation Radiation: Full-dose Radiation Drug: Temozolomide Drug: Cyclophosphamide Drug: Etoposide Drug: Lomustine	Augusta University, Georgia Cancer Center, Augusta, Georgia, United States Emory University, Children’s Healthcare of Atlanta, Druid Hills, Georgia, United States
NCT03620032	Study of Re-irradiation at Relapse Versus RT and Multiple Elective RT Courses	Drug: Nimotuzumab Drug: Vinorelbine Other: Radiotherapy	Fondazione IRCCS Istituto Nazionale Tumori, Milan, Italy
NCT03690869	REGN2810 in Pediatric Patients with Relapsed, Refractory Solid, or Central Nervous System (CNS) Tumors and Safety and Efficacy of REGN2810 in Combination with Radiotherapy in Pediatric Patients With Newly Diagnosed or Recurrent Glioma	Drug: REGN2810 (monotherapy) Drug: REGN2810 (maintenance) Radiation: Conventional or hypofractionated Radiation: Re-irradiation	Children’s Hospital Los Angeles (CHLA), Los Angeles, California, United States Rady Children’s Hospital, San Diego, California, United States UCSF Benioff Children’s Hospital, San Francisco, California, United States Children’s National Health System (Children’s National Medical Center), Washington, District of Columbia, United States University of Florida- Neurosurgery, Gainesville, Florida, United States Ann & Robert H. Lurie Children’s Hospital of Chicago, Chicago, Illinois, United States Johns Hopkins - Pediatric Oncology, Baltimore, Maryland, United States Massachusetts General Hospital, Boston, Massachusetts, United States Dana Farber Cancer Institute/ Boston Children’s Hospital, Boston, Massachusetts, United States C. S. Mott/University of Michigan, Ann Arbor, Michigan, United States and 10 more
NCT01502917	Convection-Enhanced Delivery of 124I-Omburtamab for Patients with Non-Progressive Diffuse Pontine Gliomas Previously Treated with External Beam Radiation Therapy	Drug: Radioactive iodine-labeled monoclonal antibody omburtamab Radiation: External Beam Radiotherapy	Weill Medical College of Cornell University, New York, New York, United States Memorial Sloan Kettering Cancer Center, New York, New York, United States

**Table 2:** Active clinical trials for children with diffuse intrinsic pontine glioma involving chemotherapy as found on www.Clinicaltrials.gov as of 12 January 2022.

NCT Number	Title	Intervention	Location
NCT03709680	Study of Palbociclib Combined with Chemotherapy In Pediatric Patients With Recurrent/Refractory Solid Tumors	Drug: Palbociclib Drug: Temozolomide Drug: Irinotecan Drug: Topotecan Drug: Cyclophosphamide	Children’s Hospital of Alabama, Birmingham, Alabama, United States Phoenix Children’s Hospital, Phoenix, Arizona, United States Children’s Hospital Los Angeles, Los Angeles, California, United States Children’s Hospital and Research Center at Oakland, Oakland, California, United States Children’s Hospital of Orange County, Orange, California, United States Lucile Packard Children’s Hospital, Palo Alto, California, United States University of California San Francisco, San Francisco, California, United States Children’s Hospital Colorado, Aurora, Colorado, United States Children’s National Medical Center, Washington, District of Columbia, United States and 47 more
NCT04049669	Pediatric Trial of Indoximod with Chemotherapy and Radiation for Relapsed Brain Tumors or Newly Diagnosed DIPG	Drug: Indoximod Radiation: Partial Radiation Radiation: Full-dose Radiation Drug: Temozolomide Drug: Cyclophosphamide Drug: Etoposide Drug: Lomustine	Augusta University, Georgia Cancer Center, Augusta, Georgia, United States Emory University, Children’s Healthcare of Atlanta, Druid Hills, Georgia, United States
NCT03620032	Study of Re-irradiation at Relapse versus RT and Multiple Elective RT Courses	Drug: Nimotuzumab Drug: Vinorelbine Other: Radiotherapy	Fondazione IRCCS Istituto Nazionale Tumori, Milan, Italy
NCT02992015	Gemcitabine in Newly Diagnosed DIPG	Drug: Gemcitabine	Children’s Hospital Colorado, Aurora, Colorado, United States
NCT04196413	GD2 CAR T Cells in DIPG & Spinal Diffuse Midline Glioma (DMG)	Drug: GD2 CAR T cells Drug: Fludarabine Drug: Cyclophosphamide	Lucile Packard Children’s Hospital, Stanford, California, United States
NCT04099797	C7R-GD2.CAR T Cells for Patients with GD2-expressing Brain Tumors (GAIL-B)	Drug: (C7R)-GD2.CART cells Drug: Cyclophosphamide Drug: Fludarabine	Texas Children’s Hospital, Houston, Texas, United States
NCT03396575	Brain Stem Gliomas Treated with Adoptive Cellular Therapy During Focal Radiotherapy Recovery Alone or With Dose-intensified Temozolomide (Phase I)	Biological: TTRNA-DC vaccines with GM-CSF Biological: TTRNA-xALT Drug: Cyclophosphamide + Fludarabine Lymphodepletive Conditioning Drug: Dose- Intensified TMZ Drug: Td vaccine Biological: Autologous Hematopoietic Stem Cells (HSC)	University of Florida Health Shands Children’s Hospital, Gainesville, Florida, United States
NCT03243461	International Cooperative Phase III Trial of the HIT-HGG Study Group (HIT-HGG-2013)	Drug: Temozolomide + Valproic Acid	Universitätsklinik RWTH Aachen, Aachen, Germany Klinikum Augsburg, Augsburg, Germany Charité Universitätsmedizin Berlin, Berlin, Germany HELIOS Klinikum Berlin Buch, Berlin, Germany Evangelisches Krankenhaus Bielefeld, Bielefeld, Germany Universitätsklinikum Bonn, Bonn, Germany Städtisches Klinikum Braunschweig gGmbH, Braunschweig, Germany Klinikum Bremen-Mitte gGmbH, Bremen, Germany Carl-Thiem-Klinikum Cottbus gGmbH, Cottbus, Germany Klinikum Dortmund gGmbH, Dortmund, Germany and 39 more
NCT01837862	A Phase I Study of Mebendazole for the Treatment of Pediatric Gliomas	Drug: Mebendazole Drug: Vincristine Drug: Carboplatin Drug: Temozolomide Drug: Bevacizumab Drug: Irinotecan	Cohen Children’s Medical Center of New York, New Hyde Park, New York, United States

**Table 3:** Clinical trials for children with diffuse intrinsic pontine glioma with results available as found on www.Clinicaltrials.gov as of 12 January 2022.

NCT Number	Title	Treatment Modality	Results
NCT01182350	Molecularly Determined Treatment of DIPG	Drug: Bevacizumab Drug: Erlotinib Drug: Temozolomide Radiation: RT	Study terminated early having met the pre-specified stopping rule which required 3 or more cohorts with at least 10 patients each or 2 cohorts with at least 15 patients each in the first 50 patients with informative biopsies. Treatment did not comment on OS but showed feasibility of surgical biopsy for DIPG [67].
NCT02607124	A Phase I/II Study of Ribociclib, a CDK4/6 Inhibitor, Following Radiation Therapy	Drug: Ribociclib	The study included 10 patients. The 1-year and median OS was 89% and 16.1 months. Concluded ribociclib administered following radiotherapy is feasible in DIPG but did not significantly improve OS [91].
NCT01189266	Vorinostat and Radiation Therapy Followed by Maintenance Therapy with Vorinostat in Treating Younger Patients with Newly Diagnosed DIPG	Radiation: 3-Dimensional Conformal Radiation Therapy Radiation: Intensity-Modulated Radiation Therapy Other: Laboratory Biomarker Analysis Drug: Vorinostat	This study is active but not recruiting. Study participants included 12 participants. The study showed that vorinostat given concurrently with radiation followed by vorinostat monotherapy was well tolerated in children but failed to improve a 1-year OS [92].
NCT00036569	A Phase II Study of Pegylated Interferon Alpha 2b in Children with DIPG	Procedure: adjuvant therapy Biological: pegylated interferon alfa	32 patients with DIPG enrolled in this study. The study did not significantly improve the 2-year OS in children with DIPG [89].
NCT01514201	Veliparib, Radiation Therapy and Temozolomide in Treating Younger Patients with Newly Diagnosed DIPG	Radiation: 3-Dimensional Conformal Radiation Therapy Radiation Intensity-modulated radiation therapy Other: Laboratory biomarker analysis Other: Pharmacological study Drug: Temozolomide Drug: Veliparib	66 patients were enrolled in this study. The addition of veliparib to radiation followed by TMZ was tolerated but did not improve survival for patient with DIPG with a 1- and 2-year OS of 37.2% and 5.3% [93].
NCT00879437	Valproic Acid, Radiation and Bevacizumab in Children with HGG or DIPG	Drug: Valproic acid Drug: Bevacizumab Radiation: Radiation therapy	The study enrolled 20 patients with DIPG. The addition of valproic acid and bevacizumab to radiation was well tolerated but did not improve the event free survival (7.8 months) or OS (10.3 months) in children with DIPG [94].
NCT01774253	Erivedge (Vismodegib) in the Treatment of Pediatric Patients with Refractory Pontine Glioma	Drug: Vismodegib	The study initially enrolled 9 patients but was terminated early due to lack of enrollment and given that all initial 9 patients did not complete the study.
NCT01836549	Imetelstat Sodium in Treating Younger Patients with Recurrent or Refractory Brain Tumors	Drug: imetelstat sodium	This study included a total of 42 participants and 9 with DIPG. The study was prematurely closed due to two patients suffering intratumoral hemorrhage However, no objective responses were observed within the DIPG cohort [95].
NCT03257631	A Study of Pomalidomide Monotherapy for Children and Young Adults with Recurrent or Progressive Primary Brain Tumors	Drug: Pomalidomide	The study included a total of 52 patients and 11 with DIPG. No objective response was seen in the DIPG cohort with the median OS being 5.06 months [96].
NCT02343406	Adult Study: ABT-414 Alone or ABT-414 Plus Temozolomide vs. Lomustine or Temozolomide for Recurrent Glioblastoma Pediatric Study: Evaluation of ABT-414 in Children with HGGs	Drug: Depatuxizumab mafodotin Drug: Temozolomide Drug: Lomustine	The study only included a total of six pediatric patients with high-grade gliomas with only one that completed the study. It was not specified if this patient had DIPG and their survival was not specified.

**Table 4:** Active clinical trials for children with diffuse intrinsic pontine glioma involving immunotherapeutic options as found on www.Clinicaltrials.gov as of 12 January 2022.

NCT Number	Title	Intervention	Location
NCT04749641	Neoantigen Vaccine Therapy Against H3.3-K27M DIPG	Biological: Histone H3.3-K27M Neoantigen Vaccine Therapy	Beijing Tiantan Hospital, Capital Medical University, Beijing, Beijing, China
NCT02960230	H3.3K27M Peptide Vaccine with Nivolumab for Children with Newly Diagnosed DIPG and Other Gliomas	Biological: K27M peptide Drug: Nivolumab	Rady Children’s Hospital-San Diego, San Diego, California, United States University of California San Francisco, San Francisco, California, United States Children’s National Medical Center, Washington, District of Columbia, United States Ann & Robert H. Lurie Children’s Hospital of Chicago, Chicago, Illinois, United States Dana-Farber Cancer Institute, Boston, Massachusetts, United States Children’s Hospitals and Clinics of Minnesota, Minneapolis, Minnesota, United States St. Louis Children’s Hospital, Saint Louis, Missouri, United States Nationwide Children’s Hospital, Columbus, Ohio, United States Oregon Health & Science University, Portland, Oregon, United States Children’s Hospital of Philadelphia, Philadelphia, Pennsylvania, United States and five more
NCT03396575	Brain Stem Gliomas Treated with Adoptive Cellular Therapy During Focal Radiotherapy Recovery Alone or With Dose- intensified Temozolomide (Phase I)	Biological: TTRNA- DC vaccines with GM-CSF Biological: TTRNA-xALT Drug: Cyclophosphamide + Fludarabine Lymphodepletive Conditioning Drug: Dose-Intensified TMZ Drug: Td vaccine Biological: Autologous Hematopoietic Stem Cells (HSC)	University of Florida Health Shands Children’s Hospital, Gainesville, Florida, United States
NCT04099797	C7R-GD2.CAR T Cells for Patients with GD2-expressing Brain Tumors (GAIL-B)	Drug: (C7R)-GD2.CART cells Drug: Cyclophosphamide Drug: Fludarabine	Texas Children’s Hospital, Houston, Texas, United States
NCT04196413	GD2 CAR T Cells in DIPG & Spinal Diffuse Midline Glioma (DMG)	Drug: GD2 CAR T cells Drug: Fludarabine Drug: Cyclophosphamide	Lucile Packard Children’s Hospital, Stanford, California, United States
NCT04185038	Study of B7-H3-Specific CAR T Cell Locoregional Immunotherapy for Diffuse Intrinsic Pontine Glioma/Diffuse Midline Glioma and Recurrent or Refractory Pediatric Central Nervous System Tumors	Biological: SCRI-CARB7H3(s); B7H3-specific chimeric antigen receptor (CAR) T cel	Seattle Children’s Hospital, Seattle, Washington, United States
NCT04049669	Pediatric Trial of Indoximod with Chemotherapy and Radiation for Relapsed Brain Tumors or Newly Diagnosed DIPG	Drug: Indoximod Radiation: Partial Radiation Radiation: Full-dose Radiation Drug: Temozolomide Drug: Cyclophosphamide Drug: Etoposide Drug: Lomustine	Augusta University, Georgia Cancer Center, Augusta, Georgia, United States Emory University, Children’s Healthcare of Atlanta, Druid Hills, Georgia, United States
NCT03416530	ONC201 in Pediatric *H3 K27M* Gliomas	Drug: ONC201	University of California San Francisco, San Francisco, California, United States Emory University, Children’s Healthcare of Atlanta, Druid Hills, Georgia, United States University of Miami, Miami Cancer Center, Miami, Florida, United States University of Michigan Cancer Center, Ann Arbor, Michigan, United States New York University, New York, New York, United States Cincinnati Children’s Hospital Medical Center, Cincinnati, Ohio, United States MD Anderson Cancer Center, Houston, Texas, United States Seattle Children’s Hospital, Seattle, Washington, United States
NCT04749641	Neoantigen Vaccine Therapy Against H3.3-K27M DIPG	Biological: Histone H3.3-K27M Neoantigen Vaccine Therapy	Beijing Tiantan Hospital, Capital Medical University, Beijing, Beijing, China
NCT02960230	H3.3K27M Peptide Vaccine with Nivolumab for Children with Newly Diagnosed DIPG and Other Gliomas	Biological: K27M peptide Drug: Nivolumab	Rady Children’s Hospital-San Diego, San Diego, California, United States University of California San Francisco, San Francisco, California, United States Children’s National Medical Center, Washington, District of Columbia, United States Ann & Robert H. Lurie Children’s Hospital of Chicago, Chicago, Illinois, United States Dana-Farber Cancer Institute, Boston, Massachusetts, United States Children’s Hospitals and Clinics of Minnesota, Minneapolis, Minnesota, United States St. Louis Children’s Hospital, Saint Louis, Missouri, United States Nationwide Children’s Hospital, Columbus, Ohio, United States Oregon Health & Science University, Portland, Oregon, United States Children’s Hospital of Philadelphia, Philadelphia, Pennsylvania, United States and 5 more
NCT03396575	Brain Stem Gliomas Treated with Adoptive Cellular Therapy During Focal Radiotherapy Recovery Alone or With Dose- intensified Temozolomide (Phase I)	Biological: TTRNA- DC vaccines with GM-CSF Biological: TTRNA- xALT Drug: Cyclophosphamide + Fludarabine Lymphodepletive Conditioning Drug: Dose-Intensified TMZ Drug: Td vaccine Biological: Autologous Hematopoietic Stem Cells (HSC)	University of Florida Health Shands Children’s Hospital, Gainesville, Florida, United States
NCT04099797	C7R-GD2.CAR T Cells for Patients with GD2-expressing Brain Tumors (GAIL-B)	Drug: (C7R)-GD2.CART cells Drug: Cyclophosphamide Drug: Fludarabine	Texas Children’s Hospital, Houston, Texas, United States
NCT04196413	GD2 CAR T Cells in DIPG & Spinal Diffuse Midline Glioma (DMG)	Drug: GD2 CAR T cells Drug: Fludarabine Drug: Cyclophosphamide	Lucile Packard Children’s Hospital, Stanford, California, United States
NCT04185038	Study of B7-H3-Specific CAR T Cell Locoregional Immunotherapy for Diffuse Intrinsic Pontine Glioma/Diffuse Midline Glioma and Recurrent or Refractory Pediatric Central Nervous System Tumors	Biological: SCRI- CARB7H3(s); B7H3-specific chimeric antigen receptor (CAR) T cell	Seattle Children’s Hospital, Seattle, Washington, United States
NCT04049669	Pediatric Trial of Indoximod with Chemotherapy and Radiation for Relapsed Brain Tumors or Newly Diagnosed DIPG	Drug: Indoximod Radiation: Partial Radiation Radiation: Full-dose Radiation Drug: Temozolomide Drug: Cyclophosphamide Drug: Etoposide Drug: Lomustine	Augusta University, Georgia Cancer Center, Augusta, Georgia, United States Emory University, Children’s Healthcare of Atlanta, Druid Hills, Georgia, United States

## Data Availability

Not applicable.
